# Transdiagnostic variation in age-related patterns of rich-club organization across neurodevelopmental and related mental health conditions

**DOI:** 10.1162/IMAG.a.1449

**Published:** 2026-11-10

**Authors:** Marlee M. Vandewouw, Azadeh Kushki, Russell Schachar, Elizabeth Kelley, Muhammad Ayub, Rob Nicolson, Elisabetta Trinari, Paul D. Arnold, Jason P. Lerch, Margot J. Taylor, Evdokia Anagnostou

**Affiliations:** Bloorview Research Institute, Holland Bloorview Kids Rehabilitation Hospital, Toronto, Canada; Institute of Biomedical Engineering, University of Toronto, Toronto, Canada; Autism Research Center, Bloorview Research Institute, Holland Bloorview Kids Rehabilitation Hospital, Toronto, Canada; Program in Neurosciences & Mental Health, The Hospital for Sick Children, Toronto, Canada; Department of Psychology, Queen’s University, Kingston, Canada; Department of Psychiatry, Queen’s University, Kingston, Canada; Division of Psychiatry, University College London, London, United Kingdom; Department of Psychiatry, University of Western Ontario, London, Canada; Department of Pediatrics, McMaster University, Hamilton, Canada; The Mathison Centre for Mental Health Research & Education, Hotchkiss Brain Institute, Cumming School of Medicine, University of Calgary, Calgary, Canada; Departments of Psychiatry and Medical Genetics, Cumming School of Medicine, University of Calgary, Calgary, Canada; Wellcome Centre for Integrative Neuroimaging, FMRIB, Nuffield Department of Clinical Neurosciences, University of Oxford, Oxford, United Kingdom; Department of Medical Biophysics, University of Toronto, Toronto, Canada; Diagnostic & Interventional Radiology, The Hospital for Sick Children, Toronto, Canada; Department of Psychology, University of Toronto, Toronto, Canada; Department of Medical Imaging, University of Toronto, Toronto, Canada; Institute of Medical Science, University of Toronto, Toronto, Canada; Department of Paediatrics, University of Toronto, Toronto, Canada

**Keywords:** rich-club organization, magnetoencephalography, transdiagnostic, neurodevelopmental conditions, sex differences, data-driven subgrouping

## Abstract

Rich-club organization is a topographical property of functional brain networks where highly connected regions are more densely interconnected. The rich-club has been shown to be vital for supporting cognitive functioning, and differences have been associated with a wide range of mental health and developmental conditions. This type of organization undergoes complex changes across development, and we have previously shown that males and females have strikingly distinct age-related changes. While disruptions in its development have been related to neurodevelopmental conditions and related mental health conditions such as attention-deficit/hyperactivity disorder (ADHD), autism spectrum disorder (ASD), and obsessive-compulsive disorder (OCD), the diagnostic- and sex-specific age-related patterns remain unexplored. These conditions also have heterogeneous and overlapping neurobiology and phenotype, and thus transdiagnostic approaches may be most suitable for addressing the complexities. Here, we combine the temporal advantages of magnetoencephalography (MEG) with a data-driven, transdiagnostic approach to parse the heterogeneity of age-related variation in rich-club organization in 386 neurodivergent (116 ADHD, 231 autism, 39 OCD) and 231 neurotypical children and youth, stratified by biological sex. In males and females, subgroups with distinct age-related patterns of rich-club organization did not align cleanly with the diagnostic categories. These patterns were frequency specific, separating the female subgroups in the alpha and gamma bands and the male subgroups in the theta, alpha, beta, and high gamma bands. While the female subgroups differed in their proportions of autistic and neurotypical individuals, all diagnostic categories were represented across the subgroups. The female subgroups also differed in intelligence, while the male subgroups differed in hyperactivity/impulsivity symptoms. Together, our findings indicate that frequency-specific, age-related variation in rich-club organization is largely shared across neurotypical and neurodivergent children and youth, rather than delineating diagnostic categories, and highlight the importance of considering sex when studying the biological constructs underlying neurodivergence.

## 1 Introduction

Individuals with neurodevelopmental conditions and related mental health conditions, such as autism spectrum disorder (ASD; hereafter, autism), attention-deficit/hyperactivity disorder (ADHD), and obsessive-compulsive disorder (OCD), are collectively referred to as neurodivergent, a term that captures differences in neurobiology compared with typically developing, or neurotypical, individuals. These conditions are currently defined as distinct diagnostic categories (American Psychiatric Association, 2013). These siloed labels fail to address the heterogeneity of genetic, neural, and behavioural features within conditions (Cervin et al., 2022; Lombardo et al., 2019; Sonuga-Barke et al., 2023), contributing to the lack of consensus on the nature of functional connectivity differences associated with these conditions (Cortese et al., 2021; Gürsel et al., 2018; Hull et al., 2017). The current diagnostic categories also do not consider overlapping neural and phenotypes across the conditions, with increasing evidence of shared patterns of functional connectivity across the diagnostic groups (Choi et al., 2020; Spronk et al., 2021). As such, research has shifted towards using transdiagnostic, data-driven methods to understand neurodivergence (Astle et al., 2022).

Rich-club organization, a property of functional brain networks where the most highly connected brain regions are more densely connected to one another than to other less-connected regions, is essential for the efficient transmission of information between long-range and segregated functional networks (van den Heuvel & Sporns, 2011, 2013). Reflecting its importance to information integration, the rich-club has been shown to be vital for supporting cognitive functioning, and differences have been associated with a wide range of mental health and developmental conditions (Kim & Min, 2020). For example, brain regions belonging to the rich-club are more prone to pathological alterations than to regions outside the rich-club in several neurological and psychiatric conditions (Crossley et al., 2014). Thus, studying the rich-club may provide valuable insights into the neural underpinnings of mental health.

With respect to neurodevelopmental and related conditions, differences in functional rich-club organization have been reported in ASD (Hong et al., 2019; Keown et al., 2017; Peng et al., 2023; Ray et al., 2014), ADHD (Ahmadi et al., 2021; Ray et al., 2014; Wang et al., 2021), and OCD (Peng et al., 2021) compared with their neurotypical (NT) peers, with a small study suggesting some between-diagnosis differences (Ray et al., 2014). Given that the rich-club organization of functional brain networks in typical development is known to be dependent on age and sex (Cao et al., 2014; Gozdas et al., 2019; Grayson et al., 2014; Vandewouw et al., 2023b), including age and sex as key variables is essential for characterizing the differences among these conditions. While transdiagnostic, data-driven approaches have been used to identify homogeneous subgroups of individuals with shared brain characteristics (Kushki et al., 2019, 2021; Vandewouw et al., 2023a), the construction of these subgroups has not yet considered rich-club organization nor age and sex/gender, despite their contribution to neurobiological heterogeneity in neurodivergence (Bedford et al., 2019; Carucci et al., 2023; King et al., 2019; Lombardo et al., 2019; Uddin et al., 2013).

Existing studies have used resting-state functional magnetic resonance imaging (fMRI) to measure rich-club organization in neurodivergent individuals. However, the temporal resolution of fMRI limits our ability to measure functional connectivity within the distinct oscillatory frequency bands. Given the distinct cognitive relevance, functional network organization, and development of functional connectivity within the different frequency bands (Hunt et al., 2016, 2019; Thut et al., 2012), the temporal advantages of magnetoencephalography (MEG) are better suited for capturing the full complexity of rich-club organization in neurodivergence and the associations between rich-club organization and outcomes. Indeed, using magnetoencephalography (MEG), we recently showed striking differences between males and females in the typical development of rich-club organization, and these age-related changes were specific to individual oscillatory frequency bands (Vandewouw et al., 2023b). While the temporal advantages of MEG have been leveraged to provide novel insights into differences in rich-club organization in clinical populations such as mild traumatic brain injury (Antonakakis et al., 2017a, 2017b; Rowland et al., 2017) and gliomas (Liu et al., 2020), MEG has not yet been used to investigate rich-club organization in individuals with neurodevelopmental and related conditions.

In this study, we employed a transdiagnostic, data-driven approach to investigate age-related changes in rich-club organization measured using MEG in neurodivergent (ASD, ADHD, and OCD) and NT children and youth. Using a technique called regression clustering, we identified subgroups of individuals who shared similar associations between age and rich-club organization across the oscillatory frequency bands; given our previous findings of diverging age-related associations between males and females in a normative sample, we stratified by sex. We then characterized the data-driven subgroups by their clinical characteristics. Together, this approach allows us to resolve age-related patterns of rich-club organization that are both transdiagnostic, spanning neurotypical and neurodivergent youth, and frequency specific.

## 2 Materials and Methods

### 2.1 Participants

Data were collected by the Province of Ontario Neurodevelopmental (POND) network and multiple grants awarded to MJT by the Canadian Institutes of Health Research (CIHR). Male and female participants were included in the current study if they were between 5 and 21 years of age, received a primary diagnosis of ASD, ADHD, or OCD, or who were NT, and successfully completed the resting-state MEG protocol. Previous diagnoses were confirmed using diagnostic tests and expert clinical judgement (autism: Autism Diagnostic Interview – Revised (Lord et al., 1994), Autism Diagnostic Observation Schedule – 2 (Lord et al., 2000, 2012); ADHD: Parent Interview for Child Symptoms (Ickowicz et al., 2006); OCD: Kiddie-Schedule for Affective Disorders and Schizophrenia (Kaufman et al., 1997), Children’s Yale-Brown Obsessive-Compulsive Scale (Scahill et al., 1997)). A parental screening was used to confirm typical development, defined as the absence of a premature birth, diagnoses of a neurodevelopmental or related mental health condition in the participant and first-degree family members, and any psychiatric or neurological diagnoses. The participant or primary caregiver gave written informed consent or assent, where appropriate, and the POND study was approved by all participating institutions’ research ethics boards. Data were extracted in February 2024, and 857 eligible children and youth were included in this study (158 ADHD, 330 ASD, 49 OCD, and 320 NT).

### 2.2 Demographic and phenotypic assessments

Biological sex was ascertained via self or parent report, where the respondent was asked to identify the participant’s sex at birth as female or male. Race and ethnicity data were obtained via self or parent report, where appropriate, where categories were defined according to the POND study protocol (Aboriginal, Arab, Black, Chinese, Asian, Filipino, Japanese, Korean, Latin American/Hispanic, South Asian, Southeast Asian, West Asian, White, Other, Don’t Know, Prefer not to answer). These data were used to categorize individuals into broader racial/ethnic groups (Cardenas-Iniguez & Gonzalez, 2024): Hispanic/Latino, non-Hispanic White, non-Hispanic Black, non-Hispanic Asian, Multi-racial/Other, and Prefer not to answer.

Socioeconomic status was measured using annual household income and highest caregiver education level. Annual household income was categorized into three groups: <$50,000, $50,000 to $99,999, and ≥$100,000. Caregiver education level was categorized into four groups: less than high school, high school graduate or equivalent, undergraduate, and graduate.

Medication usage was also recorded, with parents asked whether their child was currently taking any medications, and if so, to provide a list; medications were classified as anticonvulsants, antidepressants, hypnotics, non-stimulants, or stimulants. Given the limited data, the data were binarized to indicate whether an individual was taking a classified medication.

Traits characteristic of each diagnostic category was assessed as part of the POND protocol (Supplementary Table S1), defined to be social communication difficulties and repetitive behaviours for ASD, inattention and hyperactivity/impulsivity for ADHD, and obsessive-compulsive traits for OCD. Measures of intelligence were also obtained.

### 2.3 MEG data acquisition and preprocessing

Five minutes of resting-state MEG data were collected at the Hospital for Sick Children using a 151-channel CTF system (CTF-MEG International Services LP, Coquitlam, Canada) with a 600 Hz sampling rate. Participants were either presented with a fixation cross or *Inscapes*, a naturalistic movie paradigm (Vanderwal et al., 2015; Vandewouw et al., 2021a). T1-weighted MRIs were also collected to enable participant-specific head models. Fiducial coils were used to continuously track head motion, and for co-registration between the MEG and MRI data.

Using the FieldTrip MATLAB toolbox (Oostenveld et al., 2011; The Mathworks Inc., 2018), MEG data were epoched into 10 s segments, bandpass (1–150 Hz, 4^th^ order two-pass Butterworth) and notch filtered (60 Hz and the harmonics, discrete Fourier transform). Artefacts from muscle and eye movements were removed from the data using independent component analysis.

Next, epochs containing signals exceeding 2000 fT or with excessive head motion (>10 mm from the median head position) were excluded, and participants with at least 2 minutes (12 epochs) of data remaining were included in the subsequent analyses. While multiple resting-state runs (e.g., both fixation and *Inscapes*) and/or longitudinal data were collected from some participants, only one run was retained per participant. This run was chosen to ensure no significant effects of diagnosis, age, nor their interaction on the type of resting-state paradigm (Supplementary Table S2). After quality control, data from 617 participants (116 ADHD, 231 ASD, 39 OCD, and 231 NT) remained. In this sample, the number of epochs and head motion varied significantly with age and/or diagnosis (Supplementary Table S2). We, therefore, examined these variables when characterizing the data-driven subgroups.

A linearly constrained minimum variance beamformer (Van Veen et al., 1997) was used to reconstruct timeseries of neural activity for each of the 90 cortical and subcortical regions from the Automated Anatomical Labeling (AAL) atlas (Tzourio-Mazoyer et al., 2002). The MEG and MRI data were co-registered using the fiducials, and nonlinear registration was used to warp the regional coordinates from standard space into participant space. Single-shell head models were used (Nolte, 2003), and the forward solution for each coordinate was computed assuming a unit current dipole approximation, with the covariance matrix computed across all selected epochs with 5% Tikhonov regularization (Tikhonov, 1943). The data were then bandpass filtered into the five canonical frequency bands (theta: 4–7 Hz, alpha: 8–14 Hz, beta: 15–29 Hz, low gamma: 30–55 Hz, and high gamma: 65–80 Hz). To confirm these limits against the spectral structure of the data, source-level power spectra were parameterized into periodic and aperiodic components using *specparam* (Donoghue et al., 2020) over 3–45 Hz. Alpha peaks were identified within the 8–14 Hz window in the calcarine sulcus in 99.4% of participants (median: 10.66 Hz, IQR: 9.57–13.20 Hz), and beta peaks within 15–29 Hz in the precentral gyrus in 99.8% (median: 21.83 Hz, IQR: 19.08–25.25 Hz); the theta and gamma bands thus follow at the ranges below and above these limits, with gamma split into low and high by the 60 Hz line-noise notch. After bandpass filtering, data were corrected for source leakage using symmetric orthogonalization (Colclough et al., 2015), and the amplitude envelopes were computed.

Connectivity networks were constructed for each participant and frequency band by computing the amplitude envelope correlations (O’Neill et al., 2015) for pairs of brain regions. Resulting networks were thresholded using Orthogonal Minimum Spanning Trees (OMST; Dimitriadis et al., 2017) to remove spurious connections. OMST thresholding, validated using MEG functional connectivity networks (Dimitriadis et al., 2017), identifies the subset of connections such that the network efficiency and wiring cost are optimized while maintaining its connectedness. Unlike proportional thresholding, OMST thresholding is a data-driven approach that does not require the pre-specification of arbitrary parameters and produces more reliable and representative network measures than alternatives (Luppi & Stamatakis, 2021; Luppi et al., 2024; Pourmotabbed et al., 2022).

### 2.4 Rich-club organization

Rich-club organization in a connectivity network was quantified for each participant and frequency band as per our previous normative work (Vandewouw et al., 2023b). To compute the weighted rich-club coefficient (van den Heuvel & Sporns, 2011), the subnetwork with *n* connections consisting only of nodes whose degree (number of connections) exceeds a given value, *k*, is identified. The weighted rich-club coefficient at degree *k* is then computed by dividing the sum of the *n* weights in the subnetwork by the *n* largest weights in the original network. Coefficients were computed across a range of degrees, producing a curve of rich-club coefficients. The curve was normalized using 1,000 random networks (Maslov & Sneppen, 2002; Sporns & Zwi, 2004; Wang et al., 2015), and significance was assigned using the null distribution at the 5% level, corrected across the range of degrees (Benjamini & Hochberg, 1995). The area under the rich-club curve (AURC) was computed across the significant portions; values of zero indicate no rich-club organization, and larger positive values indicate more pronounced rich-club organization.

### 2.5 Regression clustering

Regression clustering was performed separately for males and females to identify subgroups of participants from their age and the band-dependent measures of rich-club organization. Following the pipeline presented in Kushki et al. (2021), Gaussian Mixture Models (GMMs) were used. GMMs assume that the data comprise subgroups that are each characterized by independent Gaussian distributions with unknown parameters; these parameters can be estimated for a pre-specified number of clusters using an expectation-maximization algorithm. To validate the appropriateness of GMMs for our data, we evaluated model fit using Akaike Information Criterion (AIC) for models with one to five subgroups (Supplementary Fig. S1). A sharp decrease in AIC from one to two subgroups, followed by diminishing improvements with additional subgroups, confirmed that the data exhibit mixture structure suitable for GMM. The regression clustering pipeline was implemented in Python (version 3.10) and consisted of three steps: (a) construction of a feature–participant matrix, where the (*i*th, *j*th) entry corresponds to participant *i* and frequency band *j*, (b) thresholding of the feature–participant matrix, and (c) clustering the feature–participant matrix.

The feature–participant matrix was constructed across 10,000 iterations, each with a randomly selected subset of participants, to increase the stability of the estimated age-related associations and reduce sensitivity to sampling variability and outliers. Given the difference in sample size between the male (*N* = 411) and female (*N* = 206) samples, a fixed subsample size—63.2% of the female sample, following standard bagging principles (Breiman, 1996)—was applied to both sexes to prevent sample-size-driven bias between the male and female analyses. This helps ensure that the clustering reflects stable, reproducible structure rather than artefacts of any single draw of the data. For each iteration, a GMM was fitted for the number of subgroups randomly selected between two and five, chosen based on our literature identifying fewer than five optimal subgroups of neurodivergent individuals with homogeneous neurobiology (Choi et al., 2020; Kushki et al., 2021; Morgado et al., 2024; Sadat-Nejad et al., 2023; Vandewouw et al., 2023a). For each subgroup, linear regression was used to investigate the associations between age and rich-club organization in each frequency band. Given the restricted childhood–adolescent age range of the sample compared with our prior lifespan neurotypical study (Vandewouw et al., 2023b), rich-club organization did not show evidence of non-linear age effects; therefore, linear models were used. If a given association was significant (*p* < 0.05), the corresponding value in the feature–participant matrix was increased. Ultimately, this resulted in a matrix whose entries corresponded to the number of times for which a participant belonged to a subgroup with a significant association between age and a given rich-club feature. Participants, therefore, group together when their rich-club organization varies with age in the same frequency bands, rather than when their rich-club values are similar.

Next, to increase validity of the feature–participant matrix by removing randomly occurring connections, the above procedure was performed 100 times with age randomly permuted. Entries of the feature–participant matrix that did not exceed the 99^th^ percentile of the entries across the random permutations were set to zero. Together, these steps enforce stability at the feature level—only associations that recur across random subsamples and exceed the permutation null are carried forward, such that the clustering operates on already-stabilized features. Finally, hierarchical clustering was performed on the thresholded feature–participant matrix, using the Euclidean distance metric and Ward linkage, producing a clustering decision tree, or dendrogram. The optimal number of subgroups was identified using the Davies–Bouldin and Calinski–Harabasz indices.

### 2.6 Statistics

Differences in the demographic and phenotypic variables between the diagnostic groups were assessed. For continuous variables, normality was first assessed using Shapiro–Wilk tests; depending on the result, one-way analyses of variance (ANOVAs) or Kruskal–Wallis tests were used, holding significance at *p* < 0.05, reporting eta-squared (η^2^) effect sizes, with post-hoc pairwise *t*- or Mann–U Whitney tests corrected using the false discovery rate (FDR) (Benjamini & Hochberg, 1995). For categorical and ordinal variables, chi-squared (*p* < 0.05, Cramer’s *v* effect sizes, and FDR-corrected pairwise chi-squared tests) and ordinal regression (*p* < 0.05, pseudo-*R*^2^ effect sizes, and post-hoc FDR-corrected pairwise *t*-tests) were used. Linear and logistic regression models were used to examine the main effects of age, diagnosis, and their interaction on the acquisition variables (number of epochs, head motion, and resting-state paradigm), with the remaining acquisition variables included as nuisance covariates.

To characterize the data-driven subgroups, age, head motion, and the number of analyzed epochs were first compared across the subgroups using the same framework. Given that all three differed (Section 3.2) in the female sample, all subsequent subgroup comparisons used regression-based models with age, head motion, and the number of analyzed epochs included as nuisance covariates: ANCOVAs (or rank-ANCOVAs when normality was violated) for continuous variables, and ordinal regression extended to include the covariates for ordinal variables. The same effect size and FDR-corrected post-hoc conventions were applied.

Linear regression models were used to test main effects of age, data-driven subgroup, and their interaction on rich-club organization, with head motion and the number of analyzed epochs as additional nuisance covariates. In the presence of a significant interaction, within-group FDR-corrected correlations (Pearson or Spearman, depending on normality) were used to determine directionality.

## 3 Results

### 3.1 Participants

Six hundred seventeen participants were included in the analysis, composed of 206 females (33 [16%] ADHD, 54 [26%] autism, 17 [8%] OCD, 102 [50%] NT) and 411 males (83 [20%] ADHD, 177 [43%] autism, 22 [5%] OCD, 129 [31%] NT). Descriptive statistics for the participant demographics by diagnosis are presented in Table 1, while the phenotypic characteristics are summarized in Supplementary Table S3. The male and female age distributions are shown in Supplementary Figure S2, and the sample characteristics are compared between sexes in Supplementary Table S4.

**Table 1.** Participant demographics for each diagnostic group, stratified by sex.

		*N* [%]				Statistics			
Females		ADHD	Autism	OCD	NT	Test statistic	*p*-value	Effect size	Post-hocs
*N*		33 [16]	54 [26]	17 [8]	102 [50]	–	–	–	–
Median age [IQR] (years)		11.1 [10.0, 12.8]	12.5 [9.8, 15.1]	12.2 [11.0, 13.3]	10.8 [8.4, 15.1]	3.36	0.340	0.01	–
Medication	No	8 [53]	16 [52]	7 [50]	21 [91]	11.20	0.011	0.37	ASD, ADHD, OCD > NT
	Yes	7 [47]	15 [48]	7 [50]	2 [9]				
Race/ethnicity	Hispanic/Latino	3 [12]	2 [6]	0 [0]	2 [8]	21.00	0.050	0.27	–
	Asian	1 [4]	5 [15]	1 [8]	5 [20]				
	Black	0 [0]	0 [0]	1 [8]	0 [0]				
	White	12 [46]	23 [70]	9 [75]	13 [52]				
	Multi-Racial/Other	10 [38]	3 [9]	1 [8]	5 [20]				
	Prefer not to answer	0 [0]	0 [0]	0 [0]	0 [0]				
Annual household income	<$50,000	3 [17]	7 [23]	0 [0]	1 [4]	3.94	0.268	0.03	–
	$50,000–$99,999	5 [28]	7 [23]	3 [50]	5 [22]				
	≥$100,000	10 [56]	16 [50]	3 [50]	17 [74]				
Highest household education	Below high school	0 [0]	0 [0]	0 [0]	0 [0]	1.96	0.582	0.01	–
	High school	2 [11]	5 [15]	1 [17]	0 [0]				
	Undergraduate	12 [67]	18 [55]	3 [50]	13 [52]				
	Graduate	4 [22]	10 [30]	2 [33]	12 [48]				
Males									
*N*		83 [20]	177 [43]	22 [5]	129 [31]	–	–	–	–
Median age [IQR] (years)		11.2 [9.7, 13.4]	12.0 [10.0, 14.7]	12.3 [9.9, 15.2]	12.0 [8.9, 14.3]	2.99	0.393	0.01	–
Medication	No	18 [41]	45 [46]	11 [61]	33 [89]	24.44	<0.001	0.35	ASD, ADHD > NT
	Yes	26 [59]	53 [54]	7 [39]	4 [11]				
Race/ethnicity	Hispanic/Latino	10 [17]	5 [6]	0 [0]	5 [12]	22.49	0.032	0.19	^1^Asian: NT > ADHD
	Asian	2 [3]	6 [7]	2 [13]	8 [20]				
	Black	0 [0]	2 [2]	0 [0]	0 [0]				
	White	29 [48]	54 [62]	7 [47]	22 [54]				
	Multi-Racial/Other	19 [32]	20 [23]	6 [40]	6 [15]				
	Prefer not to answer	0 [0]	0 [0]	0 [0]	0 [0]				
Annual household income	<$50,000	6 [20]	14 [19]	0 [0]	8 [22]	1.11	0.774	0.02	–
	$50,000–$99,999	6 [20]	22 [30]	0 [0]	8 [22]				
	≥$100,000	18 [60]	38 [51]	5 [100]	20 [56]				
Highest household education	Below high school	0 [0]	1 [1]	0 [0]	0 [0]	3.81	0.282	0.01	–
	High school	2 [6]	11 [13]	0 [0]	2 [5]				
	Undergraduate	14 [45]	45 [53]	3 [60]	24 [59]				
	Graduate	15 [48]	28 [33]	2 [40]	15 [37]				

1 No post-hoc tests were significant after correcting for multiple comparisons, and thus, uncorrected results are presented.

ADHD: attention-deficit/hyperactivity disorder; OCD: obsessive compulsive disorder; NT: neurotypical.

### 3.2 Regression clustering

For the females, the optimal number of clusters was two (Supplementary Fig. S3). The two subgroups differed significantly in age, head motion, and the number of analyzed epochs, but not in resting-state paradigm (Supplementary Table S5). As such, these variables were covaried for in all subsequent statistical comparisons.

Descriptive statistics and between-group comparisons of the phenotypic characteristics are presented in Table 2; mean connectivity networks for each subgroup are shown in Figure 1a. Subgroup-F1 consisted of 149 [72%] participants (22 [15%] ADHD, 44 [30%] autism, 14 [9%] OCD, and 69 [46%] NT), and Subgroup-F2 consisted of 57 [28%] participants (11 [19%] ADHD, 10 [18%] autism, 3 [5%] OCD, and 33 [58%] NT). The proportion of diagnostic labels differed significantly between the two subgroups (χ^2^ = 12.22, *p *= 0.007, Cramer’s *v *= 0.15), with autism overrepresented in Subgroup-F1 and NT individuals overrepresented in Subgroup-F2. Subgroup-F2 had significantly higher intelligence scores (*F* = 6.28, *p *= 0.013, η^2^ = 0.04). Note that age and intelligence were uncorrelated in the female sample (Spearman *r *= -0.03, *p *= 0.742).

**Fig. 1.**
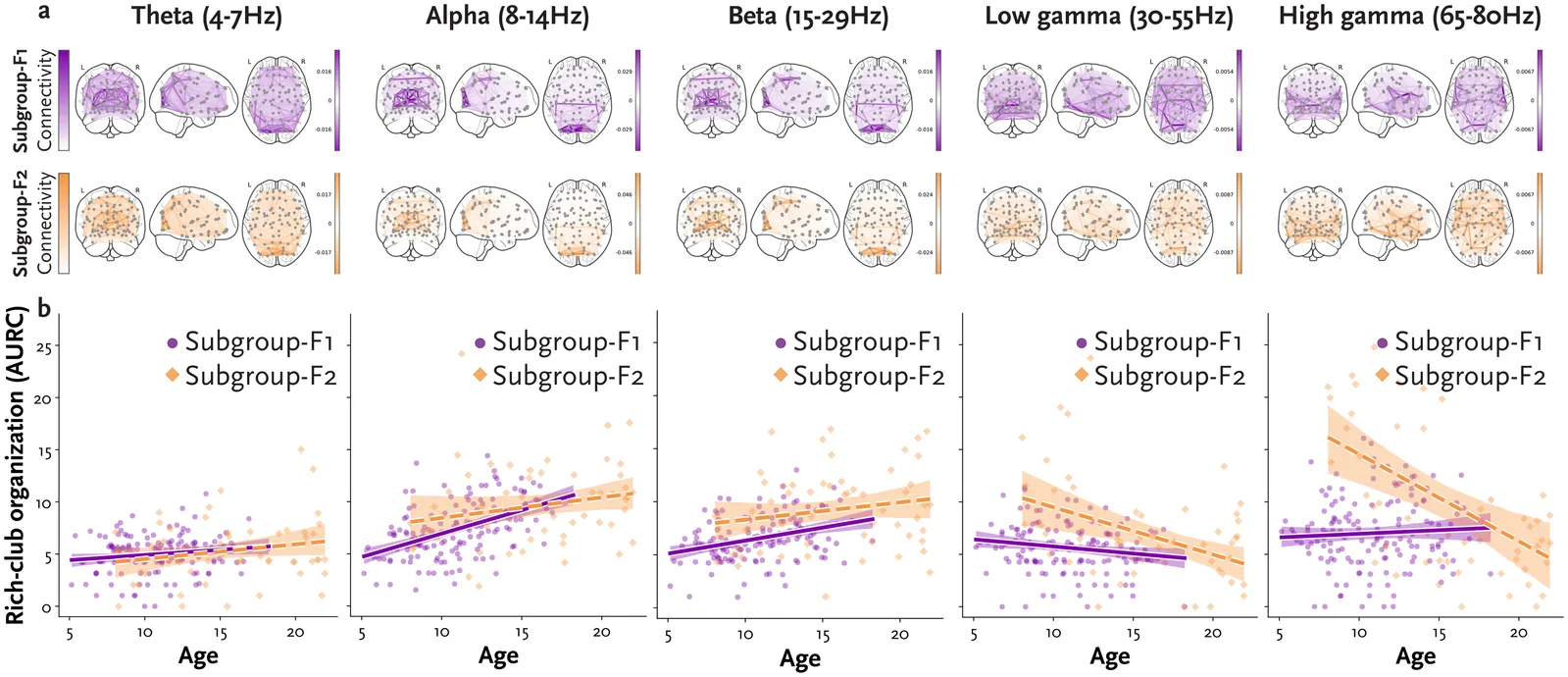
(a) Mean connectivity networks for the two female subgroups—Subgroup-F1 (purple) and Subgroup-F2 (yellow)—in each frequency band. (b) Age-related changes in each frequency band’s rich-club organization (AURC, area under the rich-club curve) in both subgroups. Shaded bands represent the 95% confidence interval around each subgroup’s regression line.

**Table 2.** Phenotypic characteristics of the female subgroups.

		N/Mean/Median [%/SD/IQR]		Statistics			
		F1	F2	Test statistic	*p*-value	Effect size	Post-hocs
*N*		149 [72]	57 [28]	–			
Dx	ADHD	22 [15]	11 [19]	12.22	0.007	0.15	ASD: F1 >F2NT: F2 > F1
	Autism	44 [30]	10 [18]				
	OCD	14 [9]	3 [5]				
	NT	69 [46]	33 [58]				
Medication	No	40 [66]	12 [55]	0.03	0.86	0.07	–
	Yes	21 [34]	10 [45]				
SCQ		5.0 [2.0, 15.0]	4.5 [1.0, 11.0]	1.13	0.292	0.01	–
RBS-R		9.0 [2.0, 25.8]	13.0 [1.2, 28.2]	0.03	0.862	<.01	–
ADHD-I		2.0 [0.0, 6.0]	1.0 [0.0, 6.0]	0.52	0.473	0.01	–
ADHD-HI		1.0 [0.0, 3.0]	0.0 [0.0, 2.2]	0.05	0.825	<.01	–
TOCS		-3.5 [-22.8, 9.5]	-3.5 [-47.2, 6.0]	1.13	0.291	0.01	–
FSIQ		107.0 [100.0, 119.0]	113.0 [106.0, 125.0]	6.28	0.013	0.04	F2 > F1

All comparisons include age, head motion, and the number of analyzed epochs as covariates.

Dx: diagnosis; ADHD: attention-deficit/hyperactivity disorder; OCD: obsessive compulsive disorder; NT: neurotypical.

Main effects of age, group, and their interaction on the measures of rich-club organization, quantified as the area under the rich-club curve (AURC), were assessed for each frequency band (Table 3; Fig. 1b). Significant age-by-group interactions were observed in alpha (*t *= -2.17, *p *= 0.031), low gamma (*t *= -2.88, *p *= 0.004), and high gamma (*t *= -5.08, *p *< 0.001). In alpha, both subgroups showed a positive association between rich-club organization and age, with more marked increases in Subgroup-F1. In low and high gamma, rich-club organization in Subgroup-F2 showed a negative association with age, while no association was observed in Subgroup-F1.

**Table 3.** Main effects of age, group, and their interaction on the measures of rich-club organization (AURC) for the females.

	Median AURC [IQR]		Statistics					
Frequency band	F1	F2	Term	B [SE]	β	*t*	*p*-value	Post-hocs
Theta (4–7 Hz)	5.4 [4.2, 6.0]	4.8 [4.2, 7.0]	Group	-0.80 [1.28]	-0.16 [0.26]	-0.63	0.53	–
			Age	0.05 [0.07]	0.08 [0.13]	0.65	0.517	–
			Group*Age	0.05 [0.09]	0.17 [0.30]	0.54	0.588	–
Alpha (8–14 Hz)	7.1 [5.6, 9.2]	9.1 [6.7, 12.3]	Group	3.98 [1.68]	0.56 [0.23]	2.37	0.019	F2 > F1
			Age	0.48 [0.09]	0.59 [0.11]	5.24	<0.001	(+) Age
			Group*Age	-0.26 [0.12]	-0.60 [0.28]	-2.17	0.031	F1, F2: (+) Age, F1 > F2
Beta (15–29 Hz)	6.3 [5.4, 7.9]	9.1 [6.2, 11.6]	Group	2.70 [1.51]	0.42 [0.23]	1.79	0.075	–
			Age	0.27 [0.08]	0.36 [0.11]	3.25	0.001	(+) Age
			Group*Age	-0.09 [0.11]	-0.23 [0.27]	-0.85	0.394	–
Low gamma (30–55 Hz)	5.8 [4.3, 7.1]	6.1 [4.3, 8.7]	Group	6.78 [1.69]	0.95 [0.24]	4.01	<0.001	F2 > F1
			Age	0.04 [0.09]	0.06 [0.11]	0.48	0.629	–
			Group*Age	-0.35 [0.12]	-0.80 [0.28]	-2.88	0.004	F2: (-) Age
High gamma (65–80 Hz)	6.6 [4.7, 9.1]	7.4 [4.4, 14.3]	Group	16.44 [2.59]	1.48 [0.23]	6.34	<0.001	F2 > F1
			Age	0.26 [0.14]	0.20 [0.11]	1.82	0.07	–
			Group*Age	-0.94 [0.18]	-1.39 [0.27]	-5.08	<0.001	F2: (-) Age

All models include head motion and the number of analyzed epochs as nuisance covariates. Post-hoc within-group correlations between age and rich-club organization are unadjusted and FDR-corrected across subgroups.

AURC: area under the rich-club curve; IQR: interquartile range; SE: standard error.

For the males, the optimal number of clusters was also two (Supplementary Fig. S3). Only the number of analyzed epochs differed significantly across the two subgroups, with age and head motion approaching significance and resting-state paradigm not differing (Supplementary Table S5). Given these trends, age, head motion, and the number of analyzed epochs were covaried for in the male analyses, consistent with the female analyses.

Descriptive statistics and between-group comparisons of the phenotypic characteristics are presented in Table 4, and mean connectivity networks for each subgroup are shown in Figure 2a. Subgroup-M1 consisted of 327 [80%] participants (70 [21%] ADHD, 137 [42%] autism, 18 [6%] OCD, and 102 [31%] NT), and Subgroup-M2 consisted of 84 [20%] participants (13 [15%] ADHD, 40 [48%] autism, 4 [5%] OCD, and 27 [32%] NT); the proportion of diagnostic labels did not differ between the subgroups. Subgroup-M2 had significantly worse hyperactivity/impulsivity symptoms compared with Subgroup-M1 (*F* = 7.90, *p *= 0.005, η^2^ = 0.04).

**Fig. 2.**
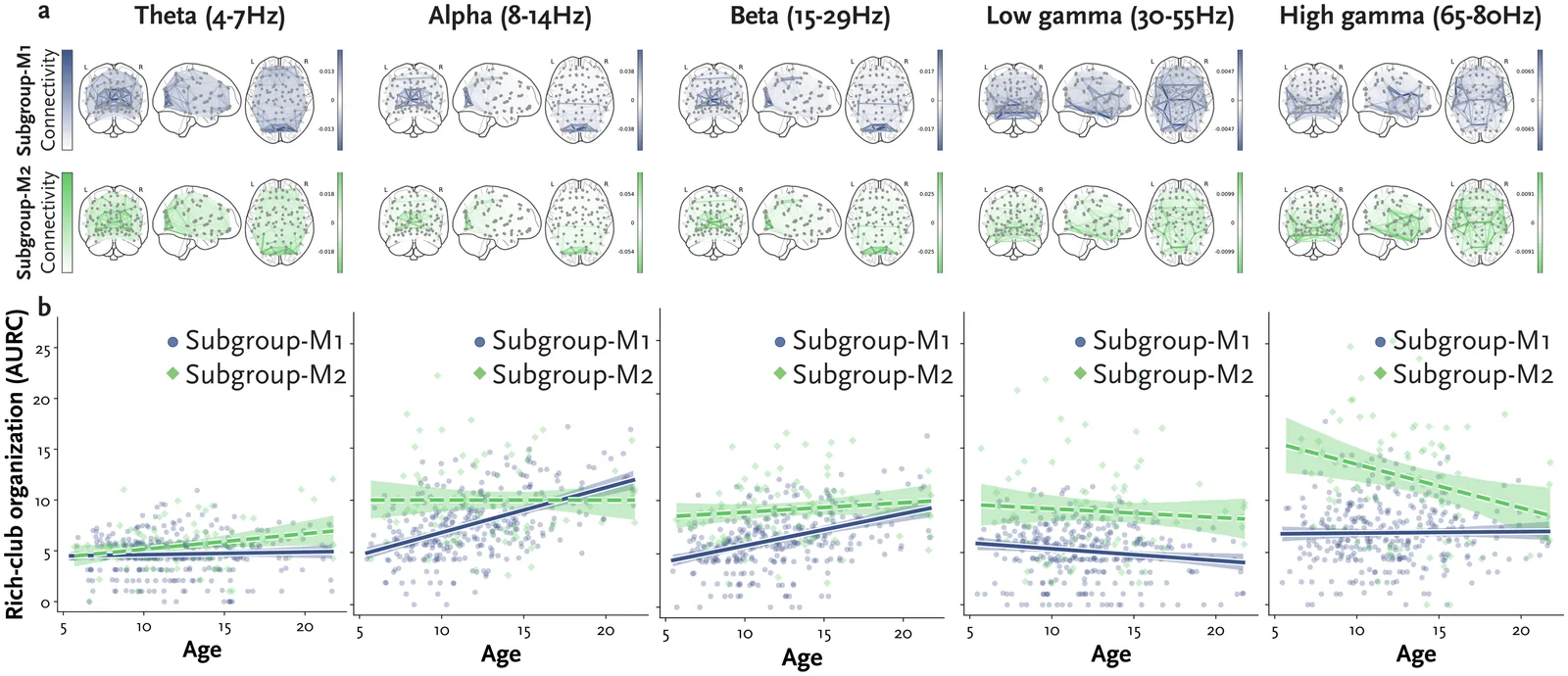
(a) Mean connectivity networks for the two male subgroups—Subgroup-M1 (blue) and Subgroup-M2 (green)—in each frequency band. (b) Age-related changes in each frequency band’s rich-club organization (AURC, area under the rich-club curve) in both subgroups. Shaded bands represent the 95% confidence interval around each subgroup’s regression line.

**Table 4.** Phenotypic characteristics of the male subgroups.

		N/Mean/Median [%/SD/IQR]		Statistics			
		M1	M2	Test statistic	*p*-value	Effect size	Post-hocs
*N*		327 [80]	84 [20]	–			
Dx	ADHD	70 [21]	13 [15]	1.77	0.621	0.07	–
	Autism	137 [42]	40 [48]				
	OCD	18 [6]	4 [5]				
	NT	102 [31]	27 [32]				
Medication	No	92 [57]	15 [41]	2.03	0.154	0.12	–
	Yes	68 [42]	22 [59]				
SCQ		10.0 [4.0, 20.0]	16.0 [9.0, 22.2]	2.32	0.129	0.01	–
RBS-R		15.0 [4.0, 30.0]	18.5 [8.0, 32.0]	0.51	0.475	<0.01	–
ADHD-I		4.0 [0.0, 7.0]	5.0 [1.0, 7.0]	0.87	0.353	<0.01	–
ADHD-HI		1.0 [0.0, 5.0]	3.0 [2.0, 6.0]	7.9	0.005	0.04	M2 > M1
TOCS		-5.5 [-42.2, 5.2]	-10.0 [-35.5, -0.8]	0.45	0.504	<0.01	–
FSIQ		107.0 [95.0, 117.0]	106.0 [93.5, 113.5]	0.16	0.688	<0.01	–

All comparisons include age, head motion, and the number of analyzed epochs as covariates.

Dx: diagnosis; ADHD: attention-deficit/hyperactivity disorder; OCD: obsessive compulsive disorder; NT: neurotypical.

For each frequency band, main effects of age, group, and their interaction on the measures of rich-club organization were examined (Table 5; Fig. 2b). Significant age-by-group interactions were observed in theta (*t* = 2.02, *p *= 0.044), alpha (*t *= -4.39, *p *< 0.001), beta (*t *= -2.43, *p *= 0.016), and high gamma (*t *= -3.36, *p *= 0.001). In theta, Subgroup-M2 showed a more positive association between age and rich-club organization than Subgroup-M1, although neither within-subgroup correlation reached significance after correction. In alpha and beta, this pattern was reversed: Subgroup-M1 showed a positive association, while Subgroup-M2 showed no association. In high gamma, Subgroup-M2 showed a negative association with age, while Subgroup-M1 showed no association.

**Table 5.** Main effects of age, group, and their interaction on the measures of rich-club organization (AURC) for the males.

	Median AURC [IQR]		Statistics					
Frequency band	M1	M2	Term	B [SE]	β	*t*	*p*-value	Post-hocs
Theta (4–7 Hz)	4.6 [3.3, 5.7]	5.5 [4.3, 6.7]	Group	-0.85 [0.88]	-0.17 [0.17]	-0.97	0.334	–
			Age	0.02 [0.03]	0.03 [0.06]	0.49	0.627	–
			Group*Age	0.13 [0.07]	0.36 [0.18]	2.02	0.044	–
Alpha (8–14 Hz)	7.6 [5.8, 9.6]	9.4 [7.5, 12.5]	Group	7.21 [1.27]	0.88 [0.15]	5.69	<0.001	M2 > M1
			Age	0.41 [0.05]	0.43 [0.05]	8.21	<0.001	(+) Age
			Group*Age	-0.42 [0.10]	-0.70 [0.16]	-4.39	<0.001	M1: (+) Age
Beta (15–29 Hz)	6.2 [4.8, 7.9]	9.2 [6.6, 10.9]	Group	5.08 [1.11]	0.71 [0.15]	4.59	<0.001	M2 > M1
			Age	0.30 [0.04]	0.36 [0.05]	6.86	<0.001	(+) Age
			Group*Age	-0.20 [0.08]	-0.38 [0.16]	-2.43	0.016	M1: (+) Age
Low gamma (30–55 Hz)	5.6 [4.2, 6.7]	8.4 [5.9, 10.9]	Group	3.28 [1.28]	0.39 [0.15]	2.57	0.011	M2 > M1
			Age	-0.05 [0.05]	-0.05 [0.05]	-1.05	0.293	–
			Group*Age	0.04 [0.10]	0.06 [0.16]	0.4	0.693	–
High gamma (65–80 Hz)	6.7 [4.8, 8.8]	12.2 [7.3, 15.6]	Group	10.40 [1.63]	0.95 [0.15]	6.39	<0.001	M2 > M1
			Age	0.09 [0.06]	0.07 [0.05]	1.43	0.154	–
			Group*Age	-0.41 [0.12]	-0.51 [0.15]	-3.36	0.001	M2: (-) Age

Post-hoc within-group correlations between age and rich-club organization are unadjusted and FDR-corrected across subgroups.

AURC: area under the rich-club curve; IQR: interquartile range; SE: standard error.

## 4 Discussion

In this study, we leveraged the oscillatory information provided by MEG to investigate rich-club organization in neurodivergent and neurotypical children and youth, using a transdiagnostic data-driven approach to identify subgroups who showed similar patterns of age-related changes. Overall, we found that subgroups with diverging patterns of rich-club organization across multiple frequency bands corresponded with phenotypic differences in both females and males. These differences were sex specific: while the data-driven female subgroups differed in intelligence scores, the male subgroups differed in hyperactivity/impulsivity symptoms. The frequency bands that distinguished the subgroups were also sex specific. In both sexes, the data-driven subgroups did not segregate cleanly along diagnostic categories. Although the female subgroups differed in diagnostic composition, all subgroups contained substantial mixing of ADHD, autism, OCD, and neurotypical individuals, suggesting overlapping rich-club organization across neurodiversity. Together, these findings point to frequency-specific patterns of age-related changes in rich-club organization that are shared across neurotypical and neurodivergent youth and that relate to phenotypic dimensions.

In both males and females, subgroups with diverging age-related patterns of rich-club organization differed in clinical phenotype. In females, the subgroups also differed in diagnostic composition: autism was overrepresented in one subgroup, and neurotypical individuals in the other. This is consistent with prior reports of altered functional rich-club organization in autism compared with neurotypical peers (Hong et al., 2019; Keown et al., 2017; Peng et al., 2023; Ray et al., 2014). Importantly, this represents partial diagnostic enrichment rather than separation—both subgroups retained substantial proportions of all four groups, and the male subgroups did not differ in diagnostic composition at all. The broader finding that subgroups defined by homogeneous neurobiology cut across diagnostic boundaries rather than aligning with them extends our previous work using structural and functional MRI (Choi et al., 2020; Jacobs et al., 2021; Kushki et al., 2019, 2021; Sadat-Nejad et al., 2023; Safar et al., 2022; Vandewouw et al., 2021b, 2023a) to MEG. This pattern suggests that the current diagnostic categories may have some, but limited, biological specificity, and is consistent with emerging models that conceptualize neurodevelopmental conditions along continuous axes rather than discrete categories. From a clinical standpoint, identifying subgroups based on shared neural mechanisms has the potential to refine classification systems and guide the development of personalized, biology-focused interventions that move beyond traditional diagnostic boundaries and better target groups with homogeneous biology rather than diagnostic label.

We found that the subgroups differed in intelligence scores and hyperactivity/impulsivity. Rich-club organization has previously been associated with intelligence (Bullmore & Sporns, 2012), and while differences have been attributed to ADHD (Ahmadi et al., 2021; Ray et al., 2014; Wang et al., 2021), we now demonstrate its association with ADHD symptoms transdiagnostically. Notably, when clustering on fMRI measures of network integration and segregation, albeit across sexes, the identified subgroups also differed in measures of intelligence and hyperactivity/impulsivity (Vandewouw et al., 2023a). Both intelligence and ADHD symptoms have been shown to engage brain-wide networks that are distributed throughout the brain, rather than local connections and/or networks (Byington et al., 2023; Mooney et al., 2024). The neural correlates of intelligence specifically have also been shown to be more readily and reliably identified than other behavioural measures, such as psychopathology (Marek et al., 2022), learning and memory (Byington et al., 2023), and executive functioning (Byington et al., 2023). This may explain why these specific phenotypic differences emerge early in the clustering decision tree (e.g., two clusters), where global differences in functional network topography tend to be captured, while local or more specific differences are refined later (e.g., five clusters).

While the female subgroups of neurodiverse children and youth with homogeneous age-related patterns of rich-club organization differed in intelligence, the male subgroups differed in hyperactivity/impulsivity. Notably, this sex-specific pattern extends to diagnostic composition: the female subgroups also differed in their proportions of autism and neurotypical individuals, whereas the male subgroups did not. This extends our previous work, which revealed sex-specific patterns of rich-club organization in typical development (Vandewouw et al., 2023b), by suggesting that these patterns contribute to downstream between-sex differences in behaviour. Sex is known to influence both biology and phenotype in neurodevelopmental conditions (see Bölte et al. (2023) for a comprehensive review), particularly in ASD and ADHD where the prevalence is unequally distributed between males and females (Posserud et al., 2021). However, sex and gender variables are seldom comprehensively examined in this population; for example, in a recent systematic review, nearly 90% of neuroimaging studies of ASD either did not explore sex whatsoever or simply controlled for it in statistical analyses (Mo et al., 2021). This work, alongside the existing evidence of sex differences in the functional brain network of neurodivergent individuals (Mo et al., 2021; Nikolaidis et al., 2022; Rosch et al., 2018; Walsh et al., 2021), highlights the importance of formally examining sex variables, as they may provide key insight into the heterogeneity of these conditions (Bölte et al., 2023).

These sex-specific patterns may reflect distinct neurobiology, differing phenotypic expression, or sample and measurement factors, which we cannot fully distinguish here. Supporting the first, the distinguishing frequency bands differed between the sexes, and our prior neurotypical work similarly found divergent age-related rich-club patterns in males and females (Vandewouw et al., 2023b). Differences in the diagnostic composition of the male and female samples may have also contributed, particularly to the narrower range of hyperactivity/impulsivity scores in the females. This account does not extend to intelligence, however, whose range was comparable across the sexes, and thus sample characteristics are less likely to be the driving factor. Hyperactivity/impulsivity is additionally recognized to present less overtly in females (Bölte et al., 2023). Disentangling these possibilities will require samples with comparable diagnostic composition across the sexes, alongside measures sensitive to female presentations of neurodivergence.

In females, the subgroups who differed in intelligence scores showed distinct age-related patterns of rich-club organization in alpha and gamma bands, with only the higher intelligence subgroup showing diverging changes in these bands. Previous MEG work has shown that a dissociation between the topography of slow and fast-scale functional networks supports intelligence in typically developing adults (Bruzzone et al., 2022). Specifically, higher intelligence was associated with increased segregation of resting-state networks in the gamma band, but increased integration in the lower frequencies (Bruzzone et al., 2022). Our work extends these findings to rich-club organization specifically, whose role is to integrate information across segregated networks (van den Heuvel & Sporns, 2013), and suggests that dissociated patterns in age-related functional network topography in low- and high-frequency bands may also support higher intelligence across neurodiversity.

However, the male subgroups with differing hyperactivity/impulsivity symptoms showed different age-related changes of rich-club organization in the theta, alpha, beta, and high gamma bands. MEG and EEG studies of ADHD thus far have predominantly focused on categorical analyses and/or inattentive traits (e.g., Lenartowicz et al., 2018), with associations between neural activity and hyperactivity/impulsivity remaining less explored and having contradictory findings (Barry et al., 2010; Kiiski et al., 2020). For example, while Barry et al. (2010) reported no significant association between hyperactivity/impulsivity and gamma activity in children, Kiiski et al. (2020) reported such an association in adults. We now show an association between hyperactivity/impulsivity traits and frequency-specific connectivity, transdiagnostically. Further work linking connectivity and ADHD traits is needed to replicate these findings, particularly with respect to measures of functional network topography.

While functional rich-club analysis is predominantly examined using fMRI, its application to MEG-derived functional connectivity captures frequency-specific network organization that haemodynamic imaging cannot access. Different oscillations reflect distinct neurophysiological processes: for example, theta and alpha oscillations facilitate long-range communication, beta oscillations support sensorimotor integration, and gamma oscillations underlie local processing (Buzsáki & Draguhn, 2004; Siegel et al., 2012). Rich-club organization within each frequency band may, therefore, reflect how hub regions coordinate information at that band’s characteristic timescale to support these distinct functions. Together, our prior (Vandewouw et al., 2023b) and present work reveal distinct age- and sex-related variation in rich-club organization, with frequency-specific patterns linked to distinct phenotypic traits, supporting the notion that hub-based network architecture is differently shaped across oscillatory timescales—and underscoring the value of frequency-resolved rich-club analysis for understanding neurodevelopment.

We would like to acknowledge that we have measured sex using a single, binarized label which does not capture the multi-categorical nature of sex and gender. Although collection of gender in the POND study is ongoing, an insufficient sample size was available for this current study; future work should examine the influence of more representative sex and gender variables on rich-club organization in both typical development and neurodevelopmental conditions. POND also uses two resting-state paradigms—fixation-cross and *Inscapes* (Vanderwal et al., 2015). While there are differences in functional connectivity between the two resting-state paradigms, we have previously shown that these differences are predominantly localized to the visual network (Vandewouw et al., 2021a), regions which are not associated with the rich-club (Grayson et al., 2014). We additionally confirmed that the type of resting-state paradigm did not differ across the data-driven subgroups. Nonetheless, the use of paradigms may still have some impact on resting-state analyses. Medication use did not differ between the data-driven subgroups in either sex; however, medication data were available for only a subset of participants, and thus residual effects, therefore, cannot be excluded. We also acknowledge that while we statistically controlled for variability in recording length, residual measurement variability associated with shorter recordings cannot be fully eliminated; future work using longer (e.g., 10-minute) resting-state acquisitions would help confirm the robustness of rich-club estimates. Although the phenotypic associations were statistically significant, they were modest in magnitude and should be interpreted in line with their effect sizes. This study examines changes in rich-club organization with age using a cross-sectional design; longitudinal analyses are required to infer cause-and-effect relationships. Other approaches, such as clustering on deviations from normative modeling, may also be better able to capture differences in age-related rich-club organization; however, normative modeling requires large sample sizes which are not yet available. Finally, while we confirmed that our frequency band limits align with the spectral structure of our data, future work characterizing age-related rich-club organization as a continuous function of frequency would establish whether these patterns depend on the band discretization used here.

An important direction for future work is the replication of these findings in independent samples. Replicability is an ongoing issue in neuroimaging studies (Poldrack et al., 2017), and given that data-driven subgrouping can be sensitive to a given cohort, independent validation is essential to rule out overfitting and to determine whether similar subgroup structures (and their associations with intelligence and hyperactivity/impulsivity) emerge in datasets with varying phenotypical profiles. At present, however, no publicly available MEG datasets include large, transdiagnostic neurodivergent samples. As such resources become available, external validation will provide a rigorous test of the stability and generalizability of rich-club-based subgroups in neurodivergent populations.

In summary, this study makes two significant contributions towards understanding the heterogeneity of the neurobiology in neurodivergence. First, we demonstrate that frequency-specific age-related patterns of rich-club organization overlap across neurotypical and neurodivergent individuals, showing partial diagnostic enrichment among females with and without autism rather than diagnostic separation, and align with transdiagnostic phenotypic characteristics. Second, age-related patterns of rich-club organization, and their association with behaviour, differ between males and females. Together, our findings advocate for sex-specific transdiagnostic approaches for understanding the biological constructs underlying neurodivergence.

## Supplementary Material

Supplementary Material

## Data Availability

Data from the POND network is available via controlled data release through the Ontario Brain Institute’s BRAIN-CODE platform. Code for the full analysis pipeline has been deposited at https://github.com/marlvan/richclub_regression_clustering/.
