# Supplementary material for: Transdiagnostic variation in age-related patterns of rich-club organization across neurodevelopmental and related mental health conditions
Source: Imaging Neurosci (Camb). 2026 Nov 10;4:IMAG.a.1449. doi: 10.1162/IMAG.a.1449 (PMC13754068; doi:10.1162/IMAG.a.1449)
Supplement: Supplementary file 1 [file IMAG.a.1449_supp.pdf]

Transdiagnostic variation in age-related patterns of rich-club organization across neurodevelopmental and related mental health conditions

Marlee M. Vandewouw[1,2], Azadeh Kushki[2,3], Russell Schachar[4], Elizabeth Kelley[5,6], Muhammad Ayub[6,7], Rob Nicolson[8], Elisabetta Trinari[9], Paul D. Arnold[10,11], Jason P. Lerch[4,12,13], Margot J. Taylor[4,14,15,16], Evdokia Anagnostou[3,17,18]

[1] Bloorview Research Institute, Holland Bloorview Kids Rehabilitation Hospital, Toronto, ON, Canada
[2] Institute of Biomedical Engineering, University of Toronto, Toronto, Canada
[3] Autism Research Center, Bloorview Research Institute, Holland Bloorview Kids Rehabilitation Hospital, Toronto, ON, Canada
[4] Program in Neurosciences & Mental Health, The Hospital for Sick Children, Toronto, Canada
[5] Department of Psychology, Queen's University, Kingston, Canada
[6] Department of Psychiatry, Queen's University, Kingston, Canada
[7] Division of Psychiatry, University College London, London, United Kingdom
[8] Department of Psychiatry, University of Western Ontario, London, Canada
[9] Department of Pediatrics, McMaster University, Hamilton, Canada
[10] The Mathison Centre for Mental Health Research & Education, Hotchkiss Brain Institute, Cumming School of Medicine, University of Calgary, Calgary, Canada
[11] Departments of Psychiatry and Medical Genetics, Cumming School of Medicine, University of Calgary, Calgary, Canada
[12] Wellcome Centre for Integrative Neuroimaging, FMRIB, Nuffield Department of Clinical Neurosciences, University of Oxford, Oxford, United Kingdom,
[13] Department of Medical Biophysics, University of Toronto, Toronto, Canada
[14] Diagnostic & Interventional Radiology, The Hospital for Sick Children, Toronto, Canada
[15] Department of Psychology, University of Toronto, Toronto, Canada
[16] Department of Medical Imaging, University of Toronto, Toronto, Canada
[17] Institute of Medical Science, University of Toronto, Toronto, Canada
[18] Department of Paediatrics, University of Toronto, Toronto, Canada

Supplemental information

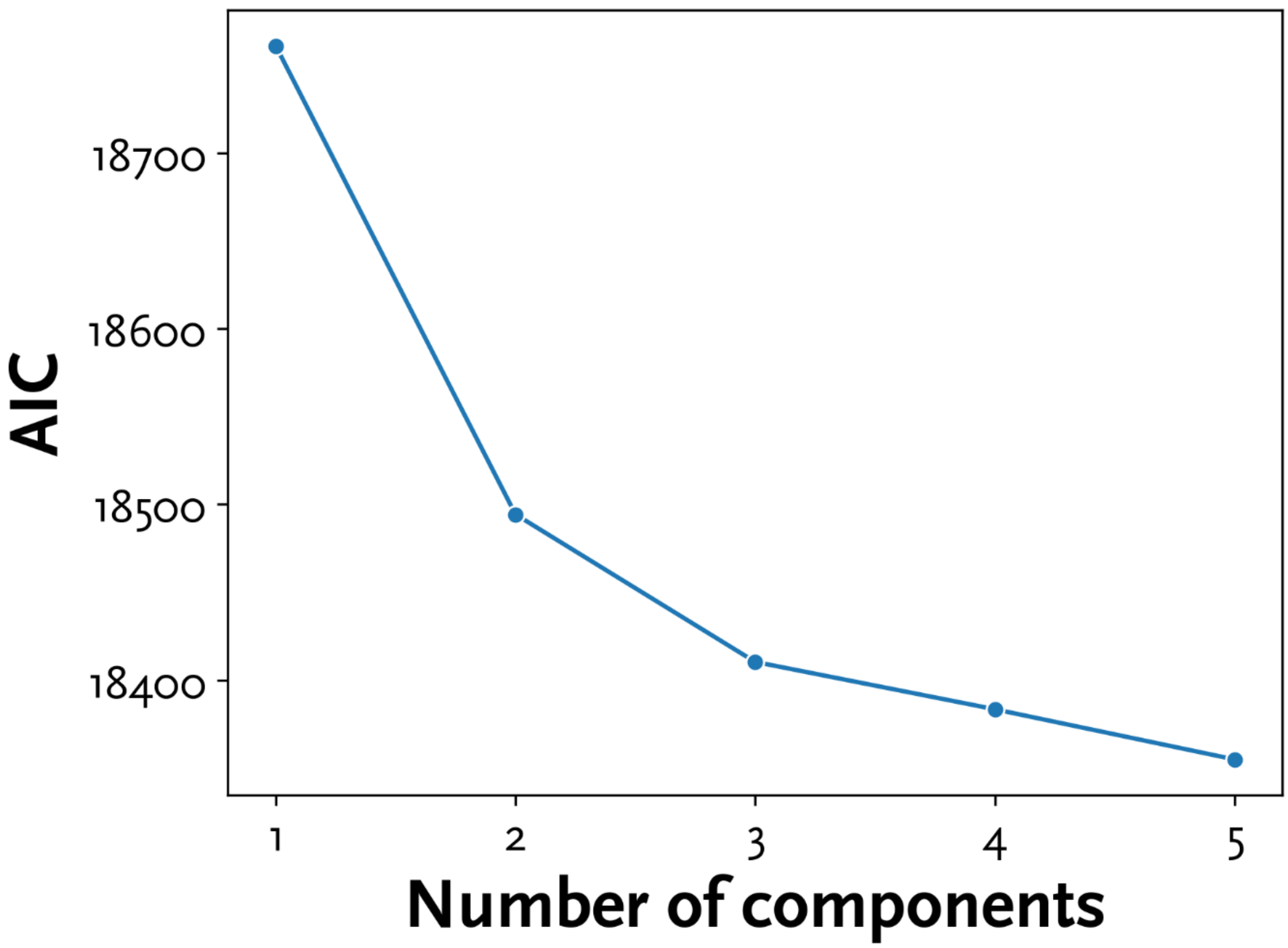

Supplemental Figure 1: Akaike Information Criteria (AIC) values for GMMs with one to five clusters, fitted to age and rich-club features across participants.

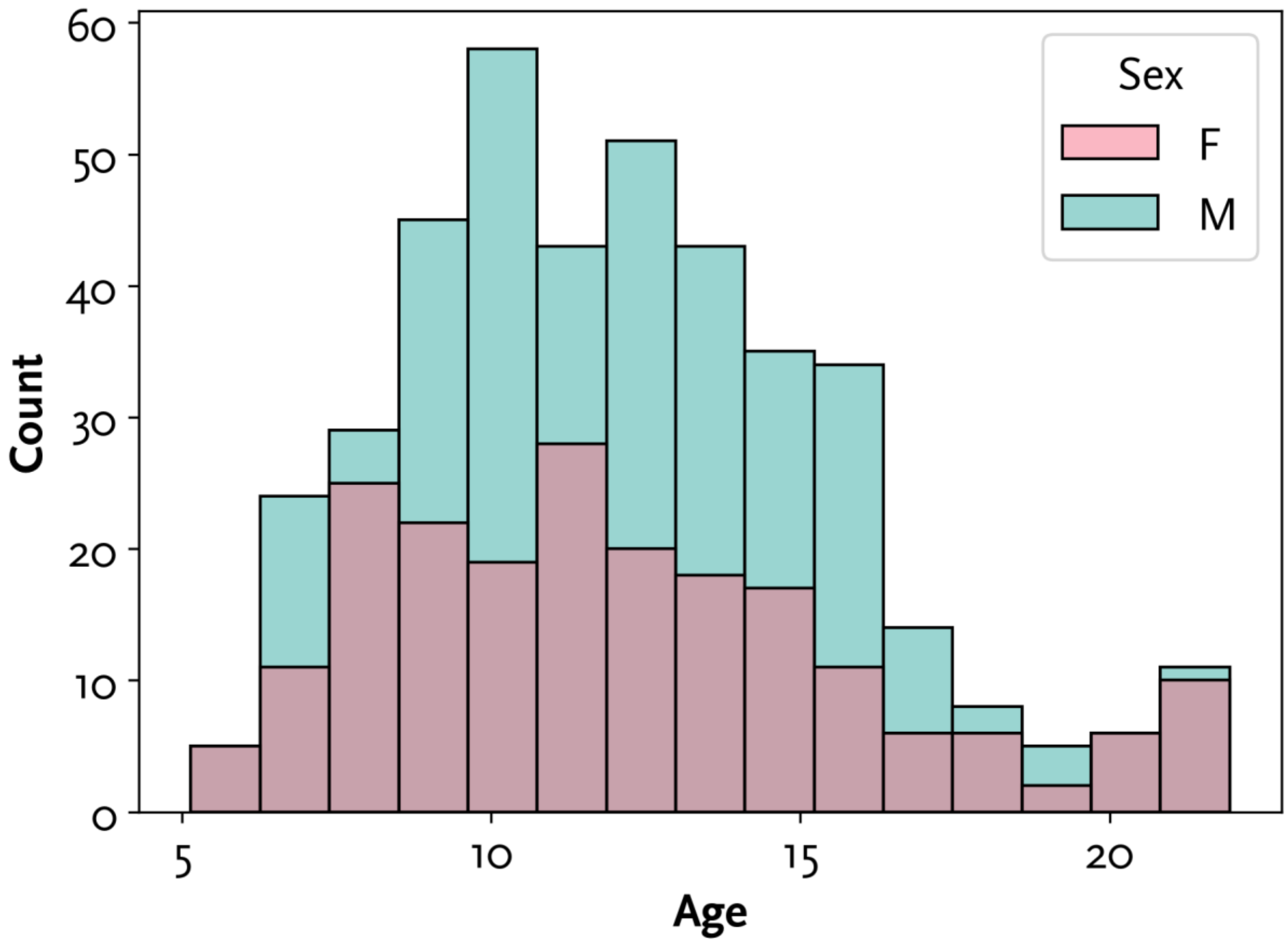

Supplemental Figure 2: Age distribution of the female (F; pink) and male (M; blue) samples.

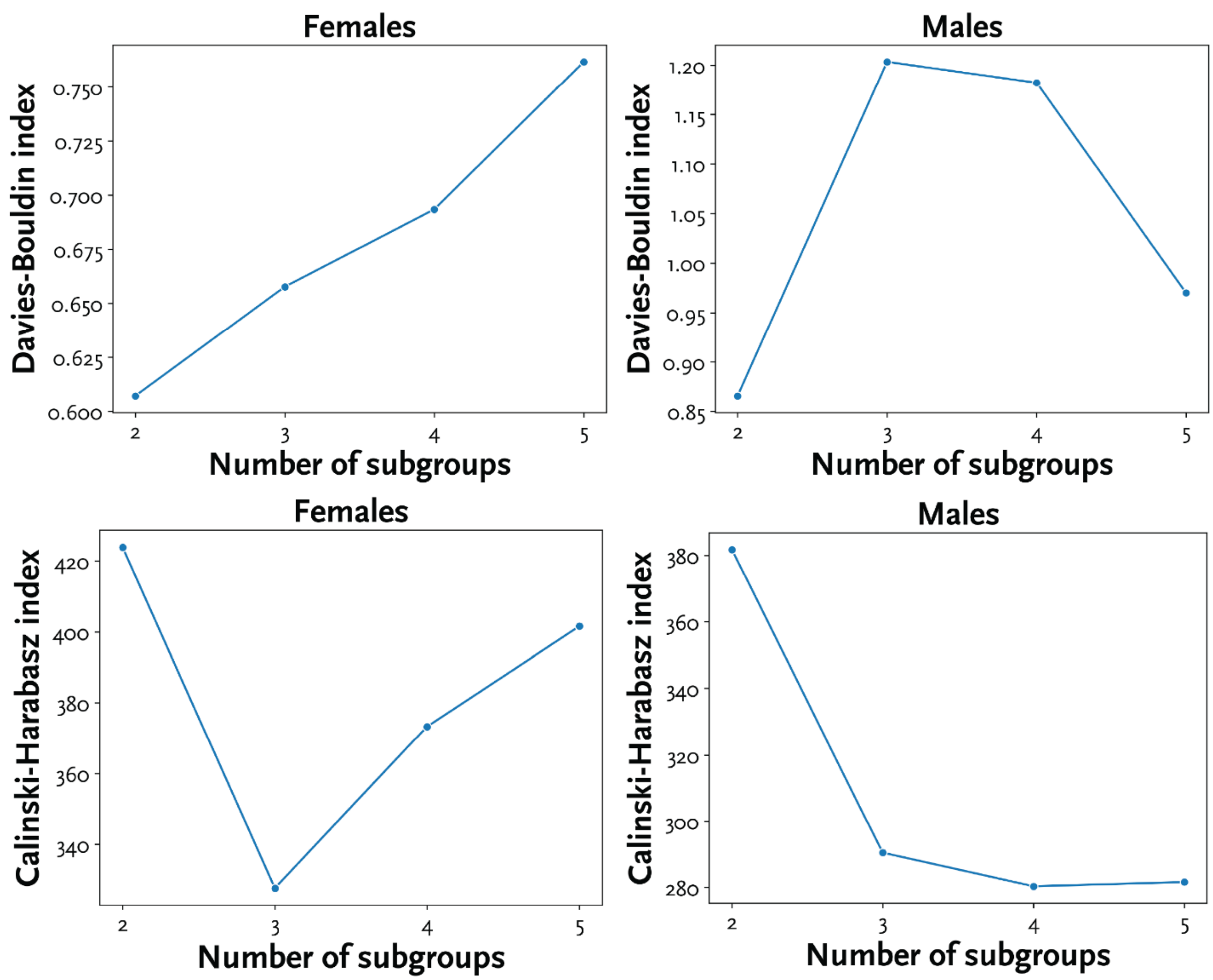

Supplemental Figure 3: Davies-Bouldin (lower values are better) and Calinski-Harabasz (higher values are better) indices for each number of clusters.

Supplemental Table 1: Summary of the assessments.

Domain	Assessment	Score	Interpretation[1]	Acronym
Social communication difficulties	Social communication questionnaire (Rutter et al., 2003)	Total score	More severe difficulties with social communication	SCQ
Repetitive behaviours	Repetitive Behaviours Scale – Revised (Bodfish et al., 2000)	Total score	More severe repetitive behaviours	RBS-R
Inattention	Strengths and Weaknesses of ADHD-symptoms and Normal Behaviour (Swanson et al., 2012)	Inattention subscale	More severe difficulties with inattention	SWAN-I
Hyperactivity and impulsivity	Strengths and Weaknesses of ADHD-symptoms and Normal Behaviour (Swanson et al., 2012)	Hyperactivity subscale	More severe difficulties with hyperactivity/impulsivity	SWAN-HI
Obsessive-compulsive traits	Toronto Obsessive-Compulsive Scale (Nelson et al., 2001)	Total score	More severe obsessive-compulsive traits	TOCS
Intelligence	Age-appropriate Wechsler or Stanford-Binet scales (Roid, 2003; Wechsler, 2014, 2011, 2003, 2002, 1999)	Full-scale intelligence quotient	Higher intelligence	FSIQ

[1]Interpretation of higher scores.

Supplemental Table 2: Descriptive statistics and regression results for the acquisition variables.

	N/Mean/Median [%/SD/IQR]				Statistics			
	ADHD	Autism	OCD	NT	Term	*F*-statistic	*p*-value	Post-hocs
Fixation<br>Inscapes	76 [66]<br>40 [34]	162 [70]<br>69 [30]	28 [72]<br>11 [28]	181 [78]<br>50 [22]	Dx<br>Age<br>Age*Dx	2.35<br>0.62<br>0.97	0.071<br>0.433<br>0.404	–<br>–<br>–
# epochs	24.0 [18.8, 27.0]	22.0 [17.0, 27.0]	23.0 [17.0, 28.5]	26.0 [20.0, 29.0]	Dx<br>Age<br>Age*Dx	3.7<br>0.01<br>0.85	0.012<br>0.935<br>0.464	–<br>–<br>–
Head motion (mm)	1.6<br>[1.1, 2.2]	1.6<br>[0.9, 2.3]	1.1<br>[0.9, 1.7]	1.1<br>[0.7, 1.7]	Dx<br>Age<br>Age*Dx	5.89<br>111.54<br>2.35	0.001<br><.001<br>0.072	ASD, ADHD > OCD<br>(-) Age<br>–

ADHD: attention-deficit/hyperactivity disorder; OCD: obsessive compulsive disorder; NT: neurotypical; Dx: diagnosis. Each model includes age, diagnosis, and their interaction, with the remaining acquisition variables as nuisance covariates.

Supplemental Table 3: Phenotypic characteristics by diagnosis, stratified by sex.

	Mean/median [SD/IQR]				Statistics			
Females	ADHD	Autism	OCD	NT	Test statistic	*p*-value	Effect size	Post-hocs
SCQ	5 [2, 11]	15 [11, 21]	2 [1, 5]	1 [0, 2]	53.97	<.001	.56	ADHD, ASD, OCD > NT ASD > ADHD, OCD
RBS-R	11 [5, 19]	25 [14, 34]	18 [7, 25]	0 [0, 2]	58.28	<.001	.37	ADHD, ASD, OCD > NT
SWAN-I	6 [5, 8]	3 [1, 6]	2 [0, 4]	0 [0, 0]	40.96	<.001	.41	ADHD, ASD, OCD > NT ADHD > ASD, OCD
SWAN-HI	5 [2, 7]	1 [0, 2]	0 [0, 2]	0 [0, 0]	44.03	<.001	.47	ADHD, ASD, OCD > NT ADHD > ASD, OCD
TOCS	-15 [-22, 4]	-3 [-19, 7]	21 [14, 34]	-41 [-57, -14]	38.74	<.001	.45	ADHD, ASD, OCD > NT OCD > ADHD, ASD
FSIQ	105 [98, 115]	101 [89, 110]	113 [106, 120]	113 [105, 124]	26.41	<.001	.17	NT > ADHD, ASD
Males								
SCQ	5 [3, 10]	20 [16, 24]	5 [3, 8]	3 [1, 4]	118.02	<.001	.56	ADHD, ASD, OCD > NT ASD > ADHD, OCD
RBS-R	11 [4, 23]	25 [16, 39]	29 [20, 35]	1 [0, 4]	134.29	<.001	.04	ADHD, ASD, OCD > NT ASD, OCD > ADHD
SWAN-I	7 [5, 8]	5 [2, 7]	1 [0, 7]	0 [0, 0]	76.22	<.001	.35	ADHD, ASD, OCD > NT ADHD > ASD, OCD
SWAN-HI	4 [1, 7]	3 [1, 6]	1 [0, 3]	0 [0, 0]	56.07	<.001	.21	ADHD, ASD, OCD > NT ADHD > OCD
TOCS	-16 [-46, -1]	-4 [-29, 3]	18 [8, 28]	-42 [-57, -7]	44.71	<.001	.22	OCD > ADHD, ASD, NT ASD > ADHD, NT
FSIQ	104 [93, 114]	100 [89, 111]	114 [98, 123]	112 [106, 120]	47.86	<.001	.15	NT > ADHD, ASD

ASD: autism spectrum disorder; ADHD: attention-deficit/hyperactivity disorder; OCD: obsessive compulsive disorder; NT: neurotypical

Supplemental Table 4: Participant demographics and phenotypic characteristics by sex.

		N/Mean/Median [%/SD/IQR]		Statistics			
		Females	Males	Test statistic	*p*-value	Effect size	Post-hocs
N		206 [33]	411 [66]	–	–	–	–
Diagnosis	ADHD	33 [16]	83 [20]	25.55	<.001	.20	Autism: Male > Female<br>NT: Female > Male
	Autism	54 [26]	177 [43]				
	OCD	17 [8]	22 [5]				
	NT	102 [50]	129 [31]				
Median age [IQR] (years)		11.7 [9.0, 14.6]	11.9 [9.6, 14.4]	0.20	.654	<.01	–
Medication	No	52 [63]	107 [54]	1.33	.249	.07	–
	Yes	31 [37]	90 [46]				
Race/ethnicity	Hispanic/Latino	7 [7]	20 [10]	2.33	.676	.09	–
	Asian	12 [12]	18 [9]				
	Black	1 [1]	2 [1]				
	White	57 [59]	112 [55]				
	Multi-Racial/Other	19 [20]	51 [25]				
	Prefer not to answer	0 [0]	0 [0]				
Annual household income	<$50,000	11 [14]	28 [19]	0.62	.430	<.01	–
	$50,000-$99,999	20 [26]	36 [25]				
	$≥100,000	46 [60]	81 [56]				
Highest household education	Below high school	0 [0]	1 [1]	0.37	.546	<.01	–
	High school	8 [10]	15 [9]				
	Undergraduate	46 [56]	86 [53]				
	Graduate	28 [34]	60 [37]				
SCQ		5 [2, 13]	12 [4, 20]	19.28	<.001	.06	Male > Female
RBS-R		10 [2, 26]	17 [5, 31]	5.77	.016	<.01	Male > Female
SWAN-I		2 [0, 6]	4 [0, 7]	7.34	.007	.02	Male > Female
SWAN-HI		0 [0, 3]	2 [0, 5]	10.70	.001	.04	Male > Female
TOCS		-4 [-27, 9]	-7 [-42, 3]	1.97	.160	.01	–
FSIQ		108 [101, 120]	107 [95, 117]	5.66	.017	.01	Female > Male

ADHD: attention-deficit/hyperactivity disorder; OCD: obsessive compulsive disorder; NT: neurotypical

Supplemental Table 5: Comparison of age, number of analyzed epochs, head motion, and resting-state paradigm across the data-driven subgroups, stratified by sex.

		N/Mean/Median [%/SD/IQR]		Statistics			
		Subgroup 1	Subgroup 2	Test statistic	*p*-value	Effect size	Post-hocs
Females	Fixation<br>Inscapes	112 [75]<br>37 [25]	39 [68]<br>18 [32]	0.65	0.422	0.06	–
	Age	10.8 [8.5, 13.2]	15.5 [11.7, 19.9]	44.86	<.001	0.29	2 > 1
	# epochs	24.0 [20.0, 28.0]	27.0 [24.0, 30.0]	7.35	0.007	0.03	2 > 1
	Head motion (mm)	1.3 [0.8, 2.3]	0.8 [0.5, 1.8]	8.46	0.004	0.02	1 > 2
Males	Fixation<br>Inscapes	230 [70]<br>97 [30]	66 [79]<br>18 [21]	1.86	0.173	0.07	–
	Age	11.7 [9.6, 14.2]	12.9 [10.0, 15.2]	3.4	0.065	0.01	–
	# epochs	22.0 [17.0, 27.0]	25.0 [21.0, 28.0]	5.17	0.023	0.02	2 > 1
	Head motion (mm)	1.4 [0.9, 2.0]	1.5 [1.0, 2.3]	3.06	0.08	0.01	–

References

Bodfish, J.W., Symons, F.J., Parker, D.E., Lewis, M.H., 2000. Varieties of repetitive behavior in autism: Comparisons to mental retardation. J. Autism Dev. Disord. 30, 237–243. https://doi.org/10.1023/A:1005596502855

Nelson, E.C., Hanna, G.L., Hudziak, J.J., Botteron, K.N., Heath, A.C., Todd, R.D., 2001. Obsessive-compulsive scale of the child behavior checklist: Specificity, sensitivity, and predictive power. Pediatrics 108. https://doi.org/10.1542/PEDS.108.1.E14

Roid, G.H., 2003. The Stanford-Binet Intelligence Scales, 5th ed. Riverside Publishing, Itasca, IL.

Rutter, M., Bailey, A., Lord, C., 2003. The social communication questionnaire. Western Psychological Services, Los Angeles, CA.

Swanson, J.M., Schuck, S., Porter, M.M., Carlson, C., Hartman, C.A., Sergeant, J.A., Clevenger, W., Wasdell, M., McCleary, R., Lakes, K., Wigal, T., 2012. Categorical and dimensional definitions and evaluations of symptoms of ADHD: History of the SNAP and the SWAN rating scales. Int. J. Educ. Psychol. Assess. 10, 51–70.

Wechsler, D., 2014. Wechsler Intelligence Scale for Children, 5th ed. The Psychological Corporation, Bloomington, MN.

Wechsler, D., 2011. Wechsler Abbreviated Scale of Intelligence, 2nd ed. NCS Pearson, San Antonio, TX.

Wechsler, D., 2003. Wechsler Intelligence Scale for Children, 4th ed. The Psychological Corporation, San Antonio, TX.

Wechsler, D., 2002. Wechsler Preschool and Primary Scales of Intelligence, 3rd ed. The Psychological Corporation, San Antonio, TX.

Wechsler, D., 1999. Wechsler Abbreviated Scale of Intelligence. The Psychological Corporation, San Antonio, TX.
